# The role of PRX1-expressing cells in periodontal regeneration and wound healing

**DOI:** 10.3389/fphys.2023.978640

**Published:** 2023-03-07

**Authors:** Zhen Huang, Xu Su, Miliya Julaiti, Xiaotao Chen, Qingxian Luan

**Affiliations:** ^1^ Beijing Key Laboratory of Digital Stomatology, NMPA Key Laboratory for Dental Materials, Department of Periodontology, National Center for Stomatology, National Clinical Research Center for Oral Diseases, National Engineering Research Center of Oral Biomaterials and Digital Medical Devices, Research Center of Engineering and Technology for Computerized Dentistry Ministry of Health, Peking University School and Hospital of Stomatology, Beijing, China; ^2^ Department of Stomatology, People’s Hospital of Xinjiang Uygur Autonomous Region, Urumqi, China

**Keywords:** paired-related homeobox protein 1, mesenchymal stem/stromal cells, wound healing, periodontal regeneration, periodontal stem cells

## Abstract

The ideal outcome of wound healing is the complete restoration of the structure and function of the original tissue. Stem cells are one of the key factors in this process. Currently, the strategy of periodontal regeneration based on mesenchymal stem cells (MSCs) is generally used to expand stem cells *in vitro* and then transplant them *in vivo*. However, their clinical application is limited. In fact, the human body has the capacity to regenerate through stem cells residing in different tissues, even without external therapeutic intervention. Stem cell niches are present in many adult tissues, such as the periodontal ligament and gingiva, and stem cells might remain in a quiescent state in their niches until they are activated in response to a regenerative need. Activated stem cells can exit the niche and proliferate, self-renew, and differentiate to regenerate original structures. Thus, harnessing the regenerative potential of endogenous stem cells *in situ* has gained increasing attention as a simpler, safer, and more applicable alternative to stem cell transplantation. Nevertheless, there are several key problems to be solved in the application of periodontal mesenchymal stem cells. Thus, animal studies will be especially important to deepen our knowledge of the *in vivo* mechanisms of mesenchymal stem cells. Studies with conditional knockout mice, in which the expression of different proteins can be eliminated in a tissue-specific manner, are especially important. Post-natal cells expressing the paired-related homeobox protein 1 (PRX1 or PRRX1), a transcription factor expressed in the mesenchyme during craniofacial and limb development, have been shown to have characteristics of skeletal stem cells. Additionally, following wounding, dermal Prx1+ cells are found out of their dermal niches and contribute to subcutaneous tissue repair. Postnatal Prx1+ cells are uniquely injury-responsive. Meanwhile, current evidence shows that Prx1+ cells contribute to promote dentin formation, wound healing of alveolar bone and formation of mouse molar and periodontal ligament. Initial result of our research group also indicates Prx1-expressing cells in bone tissue around the punch wound area of gingiva increased gradually. Collectively, this review supports the future use of PRX1 cells to stimulate their potential to play an important role in endogenous regeneration during periodontal therapy.

## 1 Introduction

Wound healing is a complex process involving many factors, the ideal outcome of which is the complete restoration of the structure and function of the original tissue. Stem cells are one of the key factors in wound healing. Mesenchymal stem cells (MSCs) are undifferentiated cells derived from tissues of the mesodermal germ layer, exist in multiple tissues and organs of the body and can differentiate into various tissues and organs (Priester et al., 2020). MSCs refer to a group of pluripotent stem cells with multiple differentiation potentials that differentiate into osteoblasts, chondroblasts, and adipoblasts. Under specific induction conditions *in vivo* and *in vitro*, MSCs can differentiate into a variety of tissue cells, such as fat, bone, cartilage, muscle, tendon, ligament, nerve, liver, myocardium, endothelium, and even blood cells. MSCs can not only differentiate into various cells, but also have various sources. However, it is still a matter of debate as to whether MSCs appear in a particular tissue because of the needs of that tissue or whether MSCs are generated and remain in these tissues during the process of development (Galipeau and Sensebe, 2018).

## 2 Challenges in the research and application of periodontal MSCs

### 2.1 Current status of MSCs research on periodontal tissues

Since the first isolation of periodontal ligament mesenchymal stem cells from human periodontal ligament (PDL) in 2004 (Seo et al., 2004), periodontal tissue regeneration therapy based on MSCs has generally involved expansion of stem cells *in vitro*, followed by their transplantation *in vivo*. However, the process of transplantation from *in vitro* culture *in vivo* is complex, high risk, and has uncertain prognosis, which limits its clinical application. In fact, the human body itself has the ability to repair tissue through stem cells resident in different tissues. Therefore, the latest treatment concept is to develop and utilize the potential of endogenous stem cells, i.e., to stimulate the body’s own stem cell potential to promote tissue healing. MSCs are found widely in adult periodontal tissue, including periodontal ligament stem cells (PDLSCs) and gingival mesenchymal stem cell (GMSCs). Like MSCs of other tissues, MSCs of periodontal tissue also have the functions of tissue regeneration and immune regulation. Usually, they exist silently in periodontal tissue and are activated only when there is a need for regeneration. Activated MSCs move from the stem cell niche, and then proliferate, self-renew, differentiate, and regenerate new periodontal tissue (Chen et al., 2011).

### 2.2 Problems encountered in MSCs study of periodontal tissue

At present, the following key problems need to be solved in the research of periodontal MSCs. Firstly, because MSCs have no obvious characteristics, the primary task is to find and determine the specific cell surface markers of MSCs in periodontal tissue, especially *in vivo* markers. Only by accurately identifying these potential MSCs can we better isolate, culture, study, and utilize them (Cui et al., 2018). Secondly, considering the good differentiation potential of MSCs, identifying MSCs from different sources of periodontal tissue is important. We should not only confirm their differentiation potential *in vitro*, but also, more importantly, confirm this pluripotency *in vivo* and expand their *in vivo* differentiation ability, especially the ability to differentiate into periodontal tissue. This should expand the indications of the diseases they treat, reduce the differences between different individuals, and improve the efficiency and direction of inducing differentiation. Therefore, in the study of periodontal MSCs, *in vivo* results are more convincing, but also more challenging (Andrukhov et al., 2019).

### 2.3 Possible solutions to the problem

Consequently, we should track the adult MSCs with regenerative potential in periodontal tissue primarily. However, previous studies on periodontal MSCs were limited by their reliance on *in vitro* detection. In 2006, the International Society for Cellular Therapy (ISCT) formulated the basic standard for identifying MSCs *in vitro* (Dominici et al., 2006). That is, the identified cells should meet following conditions. First, adherent culture ability. Second, in antigen expression, the expression of surface markers of mesenchymal cells such as CD73, CD90, and CD105 should be ≥95%, and the expression of surface markers of blood cells such as CD11b, CD14, CD34, CD45, CD19, CD79a, and HLA-DA should be ≤2%. Third, they can differentiate into osteoblasts, chondroblasts, and adipoblasts under *in vitro* induction. However, MSCs from different sources have different cell surface markers. For example, STRO-1 and CD146 [melanoma cell adhesion molecule (MCAM)] are surface markers of early MSCs, which are expressed in bone marrow and dental pulp MSCs, but do not show tissue specificity on periodontal tissue MSCs. Therefore, the general standard of MSCs cannot accurately identify periodontal tissue MSCs, and perhaps not all the so-called “periodontal stem cells” can be identified as real pluripotent periodontal MSCs. In Seo et al. (2004)’s research, PDLSCs isolated from human PDLs using STRO-1-labeled immunomagnetic beads were expanded *in vitro* and transplanted back to immunosuppressive mice, and 5/13 clones could not form mineralized or regenerate periodontal-ligament-like tissue, which indicated that not all the so-called PDLSCs identified by STRO-1 MSCs markers have regeneration potential, and false positives may occur. Thus, the ISCT MSC committee offers a new statement to clarify the nomenclature of MSCs in 2019 (Viswanathan et al., 2019). The committee continues to support the use of the acronym “MSCs” but recommends this be 1) supplemented by tissue-source origin of the cells, which would highlight tissue-specific properties; 2) intended as MSCs unless rigorous evidence for stemness exists that can be supported by both *in vitro* and *in vivo* data; and 3) associated with robust matrix of functional assays to demonstrate MSC properties, which are not generically defined but informed by the intended therapeutic mode of actions. Therefore, in PDLs, it might be necessary to add the detection of protein markers related to PDL function, such as Periostin, α-smooth muscle actin (α-SMA), and Scleraxis (SCX, a protein expressed in progenitor cells of tendons and ligaments, or differentiated cells from these ligaments). These PDL functional protein markers are also expressed in PDLSCs, and combined with the generally applicable standards of MSCs, might improve the accuracy of identifying PDLSCs (Zhu and Liang, 2015). However, even MSCs from the same site show heterogeneity. Heterogeneity of MSCs refers to the difference in the differentiation rate and level of MSCs from the same site, or the difference in the function of MSCs between different individuals. The hypothesis of heterogeneity of MSCs was first put forward around 2,000. One hundred and eighty-five human bone marrow mesenchymal stem cells (BMMSCs) were studied, and their ability to differentiate into three main cell lines, osteoblasts, chondroblasts and adipoblasts was analyzed. Except for one clone, all the other clones differentiated into osteoblasts. Accordingly, the authors proposed a hypothesis: BMMSCs with three-way differentiation potential are early mesenchymal progenitor cells, and their differentiation potential will decrease with the extension of culture time. In a follow-up study, 100 single-cell clone cultures were established and their differentiation characteristics were analyzed. It was found that the differentiation potential of these cloned cells was quite different, including three-way, two-way, and one-way differentiating clones, indicating that human BMMSCs comprise a group of cells with different differentiation potentials (Phinney et al., 1999). In addition, in the process of isolating MSCs *in vitro*, it is easy to contaminate the preparation with other types of cells, such as mixed hematopoietic cells, fibroblasts, and endothelial cells (Trubiani et al., 2019).

Therefore, to better purify and label MSCs, it is necessary to study the cell surface markers of periodontal MSCs *in vivo*, and screen out MSC subpopulations with specific functions, so that they can be preferentially differentiated into specific cell lineages, thus providing better cell sources according to the purpose of treatment. However, the study of periodontal MSCs *in vivo* is limited, and the results achieved by many *in vitro* studies using artificial intervention are difficult to realize *in vivo*. The real role of MSCs *in vivo* and their role after wound require clarification. The mechanism of multidirectional differentiation of MSCs *in vivo* is also unclear. These problems must be solved to overcome the restrictions on the clinical application of MSCs. Recently, more and more studies focus on the identification of periodontal stem cells *in vivo*. Multiple genetic markers were identified using lineage tracing methods, including Gli1, Axin2 and αSMA (Roguljic et al., 2013; Xie et al., 2019; Shalehin et al., 2022). However, since there are relatively few MSCs in PDL, it is difficult to collect MSCs from PDL. Therefore, at present, there is no consensus on the characteristics of MSCs in PDL or the mechanism of differentiation during periodontal regeneration.

## 3 Research status of PRX1 in MSCs

Paired related homeobox protein 1 (PRX1 or PRRX1) is a transcription factor expressed in mesenchymal cells in the early stage of craniomaxillofacial and limb development. It can label the progenitor cells of developing limb bones and connective tissues (Leussink et al., 1995). Prx1-deficient mice cannot survive after birth, and their skulls, limbs, and spine show severe developmental defects (Martin et al., 1995). Additionally, it was found that the expression of PRX1 was highly upregulated after amputation in salamander (Satoh et al., 2007). PRX1 is also regarded as a marker necessary to maintain the characteristics of hippocampal stem cells in adult brain tissue (Shimozaki et al., 2013). Recent cell lineage tracking studies have confirmed that PRX1-expressing cells display multipotency and can form bone and connective tissue (Gerber et al., 2018). Therefore, it is precisely because PRX1-expressing cells have this multipotent ability to form other tissues and organs that they can be used for tissue repair and reconstruction.

### 3.1 PRX1-expressing cells in bone

Bone is a highly active tissue, which is always maintained in a dynamic balance. This dynamic balance includes osteoclasts absorbing the bone matrix and osteoblasts mediating new bone formation. Osteoblasts account for 4%–6% of these osteocyte families. These cells lie on the surface of bone tissue and maintain the dynamic balance of bone. Osteoblasts are derived from osteoblast progenitors from adjacent tissues. Their main role is to produce new bone by synthesizing and assembling the intracellular matrix. The two important steps of bone matrix synthesis are the deposition and mineralization of the organic matrix: osteoblasts can secrete collagen (mainly type I collagen); and non-collagen mediated organic matrix deposition. At the same time, they mediate organic matrix mineralization through the production of alkaline phosphatase (ALP) and secretory matrix vesicles. At present, based on cell characteristics and gene expression, the downstream cells differentiated by osteoblasts at various stages have been interpreted, in which type I collagen genes (COL1A1 and COL1A2) and the ALP gene can label osteoblasts well. However, at present, we do not fully understand the identification, labeling, and localization of osteoblast upstream stem cells (Roeder et al., 2016).

Currently, studies have revealed a shift from primarily focusing on the identification of markers for postnatal skeletal progenitors to the characterization of the function of PRX1-expressing cells and their expression marker (PRX1), as a crossroad in fracture repair. The identification of fracture-induced perivascular PRX1-expressing cells and regulation of PRX1 expression by bone morphogenetic protein 2BMP2, and in turn by C-X-C motif chemokine ligand 12 (CXCL12), in the orchestration of fracture repair, highlights a pathway in which to investigate defective mechanisms and therapeutic targets for fracture non-union (Esposito et al., 2020).

Human skeletal stem cells (SSCs) are a kind of MSCs. Researchers even suggested that MSCs be named SSCs, because according to some relevant developmental evidence, after demonstrating the essence of SSCs in many aspects, they pointed out that in essence, MSCs are SSCs. Other evidence is that almost all MSCs can differentiate into osteoblasts, but not all MSCs can differentiate into adipocytes or chondrocytes. Many studies have shown that PRX1 is highly expressed in SSCs. PRX1-expressing cells in bone tissue express SSC-like characteristics and have the ability to differentiate into osteoblasts and regulate the immune response, which suggests an important role in bone regeneration. Studies have shown that MSCs expressing PRX1 in the periosteum play a significant role in bone tissue development and regeneration (Moore et al., 2018), and fracture healing (Duchamp de Lageneste et al., 2018). Clinical trials have also confirmed that implantation of such MSCs is an effective and safe treatment for defect repair.

### 3.2 PRX1-expressing cells in skin

Skin is the largest tissue of the human body, which constructs a multifunctional barrier between internal organs and the external environment. The ability of the skin to regenerate is essential to maintain skin integrity. However, adult skin wound healing often leaves scars. These fibrous scars are mainly caused by the heterogeneity of fibroblasts in the process of wound healing. In other words, the reason for the scar is that this mixed fibroblast population has internal differences. They respond differently to wound signals to reconstruct a complete structure, which causes confusion and forms a fibrotic scar. Therefore, it would be ideal to distinguish subpopulations with high regeneration or differentiation potential from these hybrid fibroblasts. These subpopulations can be isolated from fibrotic cells and can specifically recruit and expand non-fibrotic cell populations during wound healing, or conversely, they would inhibit the role of fibrotic cells in wound healing, preventing scar formation and accelerating wound healing. Studies have shown that the content of PRX1-expressing cells in adult dermal cells is very low (about 0.2%), mainly existing in the cell niches around blood vessels in the dermis and hair follicles. However, when skin wounds occur, PRX1-expressing cells rapidly migrate to the wound site and expand greatly during the healing process, participating in skin regeneration and reconstruction. Moreover, it was found that PRX1-expressing cells migrated from cell niches in the dermis are also involved in the reconstruction of subcutaneous adipocytes, fascia, and other structures (Currie et al., 2019). Therefore, the next question is, do other subtype cells in skin tissue, such as PRX1-expressing cells, expand rapidly after wounding and participate in tissue reconstruction? Further studies found that for COL1α2-labeled normal skin fibroblasts, there was no difference in the number of COL1α2-labeled cells between normal and wounded skin. By contrast, the ratio of COL1α2-expressing to PRX1-expressing cells decreased at 21 days after wounding, indicating that COL1α2-expressing cells do not expand in response to wounding. Therefore, PRX1-expressing cells are a unique cell population in adult tissues that respond to wounding (Currie et al., 2019). Moreover, in recent work using lineage tracing and single-cell transcriptomics, it was reported that PRX1-expressing cells were largely responsible for fibrosis in the ventral dermis during wound repair (Leavitt et al., 2020).

### 3.3 PRX-1 expressing cells in periodontal tissue

There are a large number of PRX1-expressing cells in the cell rich area of the dental pulp horn in mice, at 3 days or 4 weeks postpartum, which is conducive to recruiting new differentiated cells to form odontoblasts after abrasion (Wang et al., 2018). Recent studies have shown that PRX1-expressing cells also exist in the skull suture, which is necessary for the development and regeneration of the skull and the healing of skull wounds (Wilk et al., 2017). Do cells express PRX1 in periodontal tissue? Based on the structural characteristics of the alveolar fossa, where the PDL is located, which are quite similar to the skull suture, researchers have found that PRX1-expressing cells also exist in mouse and human PDLs and contribute to wound healing of alveolar bone (Bassir et al., 2019). Mouse incisors grow for the whole life of the mouse, while molars do not grow after adulthood, and the *Prx1* gene is highly related to development and regeneration. A study found that there are more PRX1-expressing cells in mouse incisor PDLs than in molar PDLs. Moreover, it was found that PRX1 continued to be highly expressed in mouse incisor PDLs at 3 and 8 weeks postpartum, while the number of PDLs cells in molar PDLs gradually decreased with increasing mouse age, which further confirmed the key role of PRX1-expressing cells in regulating growth and regeneration. In addition, the wound models were made in the alveolar bone of incisor area and molar area of mice. Under normal circumstances, they all healed 8 weeks after wounding. In the mice ablated for inducible PRX1-expressing cells, at 8 weeks after wounding, five samples of alveolar bone in the incisor area were not cured. Although three out of five samples of alveolar bone in the molar area were cured, the morphology of bone formation was irregular. In other words, after knockout of *Prx1*, alveolar bone wounds could not heal in the incisor area. Irregular bone wound healing in the molar area might reflect the role of progeny cells differentiated from residual PRX1-expressing cells (such as osteoblast precursor cells and osteoblasts). Although they can heal partially, due to the lack of pluripotent PRX1-expressing cells, these progeny cells cannot maintain a normal and orderly bone healing process, resulting in irregular healing (Bassir et al., 2019).

Although the expression of PRX1 in normal PDLs has been confirmed, there is no corresponding evidence as to whether the expression of PRX1 in PDL increases and plays a key role after wounding. In addition, it is unclear whether PRX1-expressing cells still exist in gum tissue. Gums are similar to skin and also comprise a barrier between the internal and external environment of the oral cavity. A literature review allowed us to speculate that there are PRX1-expressing cells in the gingiva, which can also quickly migrate to the wound site and expand after gingival wounding, and participate in the healing of gingival tissue. Therefore, under normal circumstances, a small number of PRX1-expressing cells exist in periodontal tissue and play a key role in wound healing, but are they MSCs? There are MSCs in PDLs, namely, PDLCs. Similarly, there are MSCs in gingiva, termed GMSCs. The study of PRX1 expression in PDLs and its involvement in alveolar bone wound healing prompted us to further question the role of PRX1-expressing cells in periodontal tissue wound healing.

## 4 Characteristics of PRX1-expressing cells and their role in wound healing

As mentioned above, several studies have confirmed that PRX1-expressing cells in bone have the characteristics of bone MSCs (Ouyang et al., 2014). Meanwhile, PRX1 is a better marker of dermal MSCs in skin than traditional platelet derived growth factor receptor alpha (PDGFRA) in skin (Currie et al., 2019). More importantly, PRX1-expressing cells found in PDL also exhibit stem cell characteristics (Bassir et al., 2019). Researchers isolated PRX1-expressing cells from mouse incisor PDLs and found that these cells expressed high levels of MSC gene markers. At the same time, in human primary PDLSCs, it was also found that the transcription level of PRX1 was significantly higher than that of MCAM and SCX. MCAM and SCX are two common markers of human PDLSCs; therefore, the expression of PRX1 can better represent human multipotent PDLSCs (Zhu and Liang, 2015).

**FIGURE 1:**
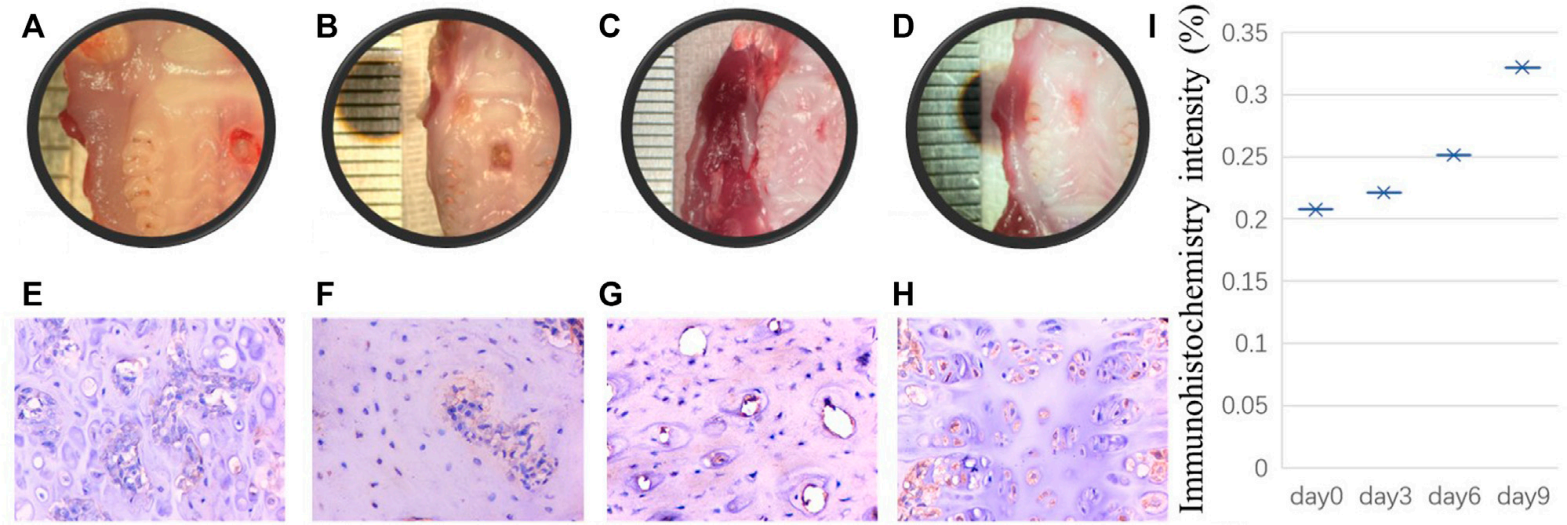
Initial results performed by our research group: A punch wound with a diameter of 2 mm was created on the palate of 8-week-old rats. The rats were sacrificed euthanized on the day of modeling, the third day, the sixth day and the ninth day after modeling respectively. Tissue samples were collected from the wound site on the day of rats sacrificed. After decalcification, the samples were embedded in paraffin, and the paraffin sections were stained for immunohistochemistry. The histological manifestations were observed and analyzed. **(A–D)** shows that punch wound healing process from day 0, 3, 6, 9 respectively. Correspondingly, for immunohistochemistry staining, **(E–H)** indicates Prx1-expressing cells in bone tissue around the defect area increased gradually from day 0**–**day 9. Scale bar, 100 µm. Immunohistochemistry intensity was calculated to reveal the difference from day 0, 3, 6, and 9 in ImageJ **(I)**.

It is unclear whether PRX1-expressing cells play a role in the tissue healing process of periodontal tissue wound sites. Previous studies on skin and PDLs did not isolate PRX1-expressing cells from the wound site for *in vitro* research. It is unclear whether the characteristics of PRX1-expressing cells after trauma are the same as those of PRX1-expressing cells under normal conditions. However, the observation of real-time dynamic images of connective tissue during the regeneration of salamander limbs confirmed that cell migration plays a key role (Currie et al., 2016). The high expression of PRX1 in the regeneration area led people to speculate that PRX1 affects cell migration, because it has been proven to affect cell migration downstream of focal adhesion kinase (FAK) (McKean et al., 2003). The high expression of *Itgb1* (encoding integrin subunit beta 1) and *Foxo3* (encoding forkhead box O3) genes, which are related to stem cell homing and colonization in PRX1-expressing cells of the mouse PDL, might provide some evidence for this speculation (Bassir et al., 2019). Moreover, a recent study (Gong et al., 2022) has provided evidence that PRX1-expressing cells are a key subtype of dental MSCs involved in the formation of mouse molar and PDL, and participate PDL reconstruction after tooth transplantation. They found that PDL formation is associated with a high number of PRX1-expressing cells; during transplanted teeth PDL reconstruction, PRX1-expressing cells from the recipient alveolar bone participate in angiogenesis as pericytes. Overall, PRX1-expressing cells are a key subtype of dental MSCs involved in the formation of mouse molar and PDL and participate in angiogenesis as pericytes during PDL reconstruction after tooth transplantation. To evaluate the role of PRX1-expressing cells in periodontal wound healing, our research group also conduct a series experiment recently. By using of gingival wound mouse model, our initial result shows that during gingival wound healing, Prx1-expressing cells in gingiva and bone tissue around the defect area increased gradually (Figure 1). Therefore, current evidence indicates that PRX1-expressing cells are present within the periodontal tissue and express MSC-like characters. They expand rapidly upon wound healing and contribute to the periodontal regeneration process.

## 5 Conclusion

In conclusion, promoting periodontal tissue healing is a popular topic in periodontal research, in which research on periodontal stem cells is a key point. Limited by the application of exogenous stem cells, stimulating endogenous MSCs provides a new idea for periodontal wound healing. A literature review indicated that the expression of PRX1 in the periosteum can locate MSCs *in vivo*. MSCs expressing PRX1 play a significant role in bone tissue development and fracture. At the same time, PRX1-expressing cells are a unique cell population in skin tissue in response to wounding. PRX1-expressing cells also exist in PDLs (Table 1). It is likely that PRX1-expressing cells also exist in other periodontal tissues, such as gingiva, playing a key role in periodontal wound healing and tissue regeneration. However, there are some bottleneck problems that still exist, such as the lack of *in vivo* mechanism research on tissue regeneration using mesenchymal stem cells, and the lack of purification and labeling methods of periodontal mesenchymal stem cell subsets. By exploring the fates of PRX1-expressing cells in target site using a lineage tracing mouse model and single-cell sequencing, combined with immunofluorescence assay might be provided as the potential solutions.

**TABLE 1:** Milestones of PRX1 cells in tissue regeneration.

Present in	Contribute to	Model	Study
Migrating fibroblasts on the ventral surface	Limb regeneration	Amphibian	McKean et al. (2003)
Periosteum of long bones	Post-natal bone development and fracture repair	Mouse	Moore et al. (2018); Duchamp de Lageneste et al. (2018)
Calvarial synarthroses	Calvarial bone regeneration	Mouse	Wilk et al. (2017)
Dermis perivascular and hair follicle	Injury healing	Mouse	Currie et al. (2019)
Dental pulp horn	Promoting dentin formation	Mouse	Wang et al. (2018)
Incisor PDL	Wound healing of alveolar bone	Mouse	Bassir et al. (2019)
Molar PDL	Formation of mouse molar and PDL; participate in PDL reconstruction after tooth transplantation	Mouse	Gong et al. (2022)
